# Supplementary material for: Quantitative Trait Loci for Heat Stress Tolerance in Brassica rapa L. Are Distributed across the Genome and Occur in Diverse Genetic Groups, Flowering Phenologies and Morphotypes
Source: Genes (Basel). 2022 Feb 3;13(2):296. doi: 10.3390/genes13020296 (PMC8871826; doi:10.3390/genes13020296)

Figure S1. Comparison of six models for variation of observed *p* value (x-axes) against the expected *p* values (y-axes) using general linear model (GLM) and mixed linear model (MLM).

GLM: QQ plots

(i) Naïve model (control)

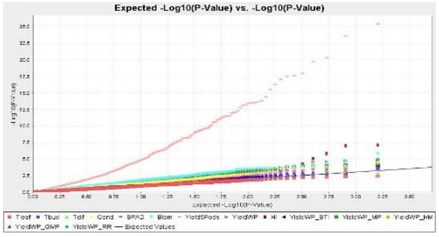

Naïve model (heat stress)

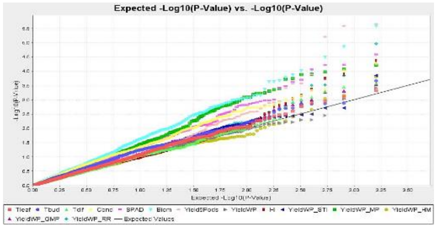

(ii) Q model (control)

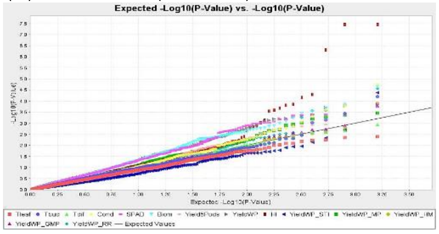

Q model (heat stress)

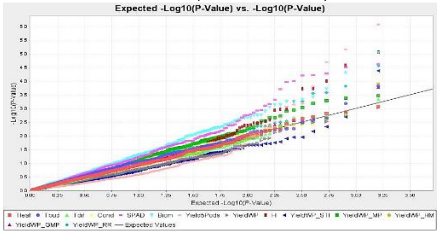

(iii) P model (control)

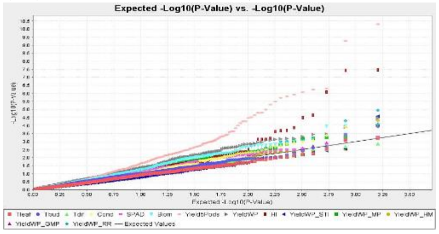

P model (heat stress)

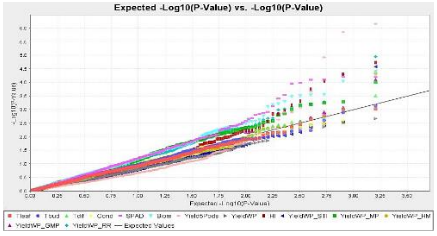

MLM: QQ plots

(iv) K model (control)

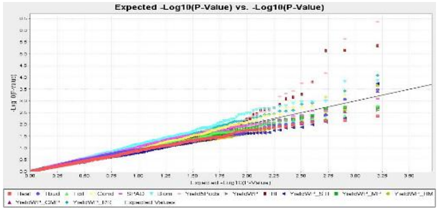

K model (heat stress)

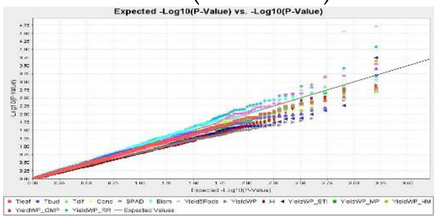

(v) Q+K model (control)

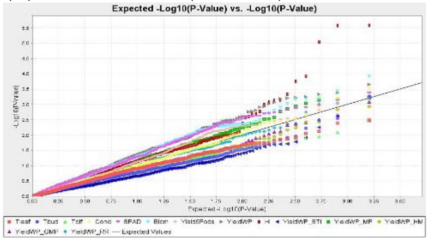

Q+K model (heat stress)

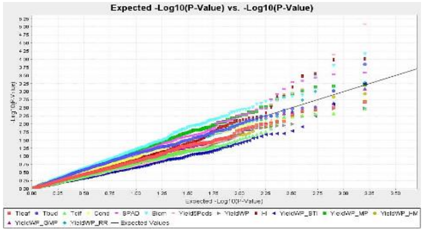

(vi) P+K model (control)

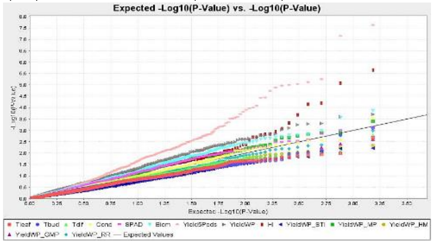

P+K model (heat stress)

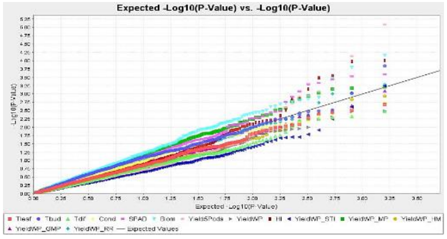

Supplement: Supplementary file 1 [file 2022_0108 Supplementary Figure S1.pdf › 2022_0108 Supplementary Figure S1.pdf]
